# Elevated anti-Zta IgG levels and EBV viral load are associated with site of tumor presentation in endemic Burkitt's lymphoma patients: a case control study

**DOI:** 10.1186/1750-9378-5-13

**Published:** 2010-07-28

**Authors:** Amolo S Asito, Erwan Piriou, Peter Sumba Odada, Nancy Fiore, Jaap M Middeldorp, Carole Long, Sheetij Dutta, David E Lanar, Walter G Jura, Collins Ouma, Juliana A Otieno, Ann M Moormann, Rosemary Rochford

**Affiliations:** 1 Maseno University, Maseno, Kenya; 2 Center for Global Health Research, Kenya Medical Research Institute, Kisumu, Kenya; 3 SUNY Upstate Medical University, Syracuse, NY, USA; 4 VU University Medical Center, Amsterdam, The Netherlands; 5 National Institutes of Health, Bethesda, MD, USA; 6 Walter Reed Army Institute of Research, Silver Springs, MD, USA; 7 Kenya Ministry of Health, Kisumu, Kenya; 8 University of Massachusetts, Worcester, MA, USA

## Abstract

**Background:**

Endemic Burkitt's lymphoma (BL) is an extranodal tumor appearing predominantly in the jaw in younger children while abdominal tumors predominate with increasing age. Previous studies have identified elevated levels of antibodies to *Plasmodium falciparum *schizont extracts and Epstein-Barr virus (EBV) viral capsid antigens (VCA) in endemic BL relative to malaria exposed controls. However, these studies have neither determined if there were any differences based on the site of clinical presentation of the tumor nor examined a broader panel of EBV and *P. falciparum *antigens.

**Methods:**

We used a suspension bead Luminex assay to measure the IgG levels against EBV antigens, VCA, EAd, EBNA-1 and Zta as well as *P. falciparum *MSP-1, LSA-1, and AMA-1 antigens in children with BL (n = 32) and in population-based age-and sex-matched controls (n = 25) from a malaria endemic region in Western Kenya with high incidence of BL. EBV viral load in plasma was determined by quantitative PCR.

**Results:**

Relative to healthy controls, BL patients had significantly increased anti-Zta (*p *= 0.0017) and VCA IgG levels (*p *< 0.0001) and plasma EBV viral loads (*p *< 0.0001). In contrast, comparable IgG levels to all *P. falciparum *antigens tested were observed in BL patients compared to controls. Interestingly, when we grouped BL patients into those presenting with abdominal tumors or with jaw tumors, we observed significantly higher levels of anti-Zta IgG levels (*p *< 0.0065) and plasma EBV viral loads (*p *< 0.033) in patients with abdominal tumors compared to patients with jaw tumors.

**Conclusion:**

Elevated antibodies to Zta and elevated plasma EBV viral load could be relevant biomarkers for BL and could also be used to confirm BL presenting in the abdominal region.

## Background

Endemic Burkitt's lymphoma (BL) is the most common pediatric cancer in areas that experience stable *Plasmodium falciparum *transmission, and both Epstein-Barr virus infection (EBV) and holoendemic *P. falciparum *are thought to be etiologically linked to this B cell neoplasm [1-4]. BL is an aggressive extranodal B cell lymphoma that can present in a number of different sites including jaw, abdomen, central nervous system, thyroid gland, orbital, and breast or a combination of these areas [5,6]. However, jaw and abdominal tumors are the most common sites of presentation. Interestingly, there are different epidemiologic patterns associated with children presenting with jaw compared to abdominal tumors. For example, while the median age of onset is 6 to 7 years for BL [7,8], jaw tumors are associated with a younger age of presentation and more frequently found in males, while abdominal tumors are common in older children, and there is a more equal distribution among males and females [6]. Despite the fact that endemic BL has several distinct clinical pictures, most studies have looked at endemic BL as a single clinical entity.

The EBV life cycle comprises both lytic and latent phases, both of which play a role in the pathogenesis of EBV-associated malignancies, and induce different antibody responses [4,9]. EBV nuclear antigen (EBNA)-1 is a latent antigen and is the only latent antigen consistently expressed in BL tumors [10]. The EBV immediate early lytic transcript BZLF1 which encodes the Zta protein can also be detected in a subset of BL tumors [11,12] but whether elevated levels of antibodies against Zta occur in children with BL is unknown. Two additional lytic antigens, viral capsid antigen (VCA) and early antigen (EAd) have been used as serologic markers of past EBV infection (e.g. VCA IgG) or viral reactivation (e.g. EAd IgG) [13]. Seminal studies in Uganda showed that elevated titers of IgG against VCA were a prognostic risk factor for BL development [14]. In addition, two recent case control studies in Uganda and Malawi have found that children with BL are more likely to have elevated antibody titers against VCA as compared to controls [8,15]. These same studies also reported elevated IgG antibody titers against *P. falciparum *schizont extracts in BL patients compared to controls [8,15].

Until recently, measurement of pathogen-specific antibodies was done with indirect immunofluorescence assays or ELISA assays. These assays have the disadvantage of allowing the measurement of only one antibody:antigen complex per sample, which can otherwise restrict the study of antibodies to multiple proteins when sample volumes are limited. In order to overcome this problem, multiplexed serology has recently been developed for the study of antibodies to multiple antigens within the same sample [16-18]. This assay can analyze more than 100 analytes simultaneously from a small sample volume enabling not only mass screening of several antigens, but also studies in pediatric populations with limited sample volumes [17,18]. In addition, this assay is less laborious and has fewer reaction steps compared to the traditional ELISA [17] and the results are highly reproducible [18]. We have recently developed a Luminex-based suspension bead assay, allowing the determination of levels of IgG to four different EBV antigens (EBNA, VCA, EAd and Zta) within the same sample [16]. In this study, we used this technology to enable more detailed study of antibody responses to an increased panel of antigens in BL patients in an age-and sex-matched controls living in a region where the risk of BL is high [7]. The panel included *P. falciparum *blood stage antigens (apical merozoite antigen (AMA)-1, merozoite surface protein (MSP)-1 that were specific for two strains of *P. falciparum *(3D7 and FV0) and a liver stage antigen (LSA)-1, in addition to the earlier mentioned EBV antigens. In addition, we also measured EBV viral load in the plasma. We report differences in both EBV serology and viral load profiles in children with BL compared to control children with the unexpected finding that the clinical presentation of the tumor correlated with levels of EBV Zta antibody and plasma viral load.

## Results

### Study participant demographic characteristics

Children with endemic BL were recruited at admission to Nyanza Provincial General Hospital (NPGH), the major referral hospital for cancer treatment in Nyanza province, Kenya. Children admitted to this hospital for reasons other than cancer typically are from Kisumu city. However, children with BL tend to come from rural areas [7] and this was also true in the current study. Therefore, our control population for this study was from a rural area with high BL incidence and persistent *P. falciparum *malaria transmission [7]. The demographic and clinical characteristics of the study participants are presented in Table 1. There were 32 children with BL and 25 controls frequency matched for age and gender. The mean age of the control children was 7.6 years and the mean age of the BL patients was 7.5 years. There were 21 male and 11 female children with BL (66% male) and 16 males and 9 females (64% male) in the control group of children. Of the BL patients, there were 14 that exclusively presented with tumor in the jaw and 16 that had only abdominal tumors. In addition, there was 1 patient that had both jaw and abdominal tumors and 1 patient with a lower left limb tumor. When the BL patients were stratified into clinical groups based on site of tumor presentation, children presenting with jaw tumors were younger [mean age, 5.6 years (IQR, 3.8-6.5)] than those presenting with abdominal tumors [mean age, 9.2 years (IQR, 6.3-11.00)] (*p *= 0.0018). Elevated serum lactate dehydrogenase (LDH) is used clinically as a marker of overall tumor burden [19]. Interestingly, BL patients with abdominal tumors had significantly higher LDH levels compared to patients with facial tumors (*p *= 0.0394).

**Table 1:** The demographic and clinical characteristics of the study participants

Parameters	Controls (n = 25)	BL (n = 32)	P value^a^	BL with abdominal (n = 16)	BL with jaw (n = 14)	P value^a^
Mean age years [range]*	7.6[5.5-10]	7.5[5-11]	n.s.	9.2[6.3-11]	5.6[3.8-6.50]	0.0018
Gender n (% male)	16(64)	21(66)	n.s.	11(79)	9(56)	n.s
Parasitemia n (% positive)	17(68)	4(13)	< 0.0001	4(29)	0(0)	< 0.0001
LDH (U/l) [SEM] **	467 (19.1)	1378(165.1)	< 0.0001	1716(236.2)	994.6(231.3)	< 0.0001
EBV viral load (Log copies/ml)[SEM]	.996 (0.36)	3.552(0.26)	< 0.0001	3.32(0.308)	3.81(0.407)	0.0483

*Data are presented as medians (in brackets are the 25^th ^and 75^th ^percentiles). ^a ^Mann-Whitney test was used to determine differences in medians between controls and BL patients and between patients with jaw and abdominal tumors. n.s denotes non significant *p *values. Abbreviation: BL, Burkitt's Lymphoma; LDH, lactose dehydrogenase; SEM, standard error of mean. **n = 30 BL patients

### BL patients and controls from malaria endemic region have comparable levels of IgG against *P. falciparum *antigens

**Figure 1:**
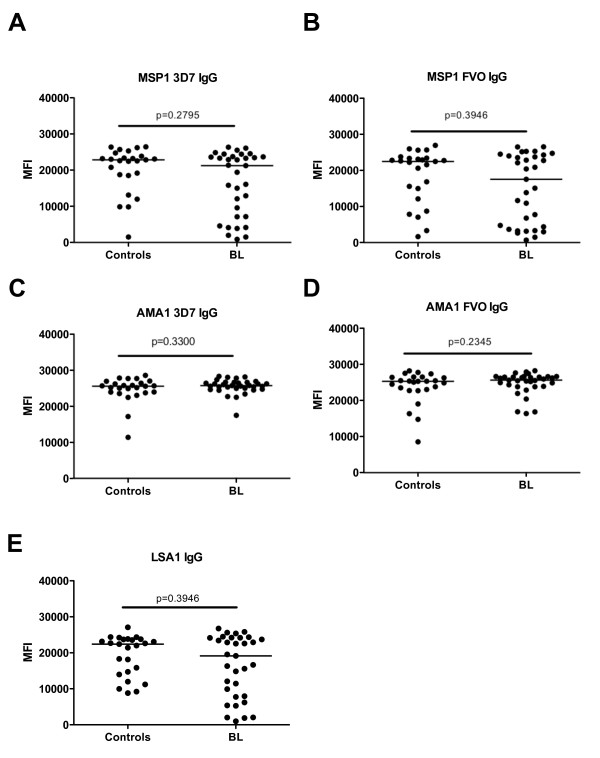
***P. falciparum*-specific IgG in controls (n = 25) versus children presenting with BL (n = 32)**. Plasma was diluted at 1:6400 and tested using a Luminex Bead Based array. Specific malaria antigens were (A) MSP1 3D7-specific IgG, (B) MSP1 FVO-specific IgG, (C) AMAI 3D7-specific IgG, (D) AMA1 FVO-specific IgG and (E) LSA1-specific IgG. The mean flourescence intensity (MFI) of 75 of Luminex beads for each of the antigen tested is indicated on the y-axis. *P *values of Mann-Whitney U tests are indicated in the figures. Horizontal bars represent median values per each study population.

To assess antibody levels to a broad panel of purified *P. falciparum *antigens, we used a Luminex bead-based array assay to measure IgG specific for 3 different malaria antigens: AMA-1, MSP-1 and LSA-1. In addition, we analyzed AMA-1 and MSP-1 antigens derived from 2 different known circulating strains of *P. falciparum*, the 3D7 and FVO strains. We detected antibodies against the malaria antigens tested in all the patients and controls (Fig. 1). Remarkably, the relative levels of IgG against all *P. falciparum *antigens tested were comparable between BL patients and controls and no significant differences were detected. Sixty-eight percent of the controls had *P. falciparum *parasites in their blood while only 13% of the BL patients were *P. falciparum *positive (Table 1). However, most children presenting with BL at NPGH have been referred from another hospital, and in most cases it was reported that these patients have been treated with anti-malarial drugs prior to admission at NPGH.

### BL patients have increased EBV-specific Zta and VCA IgG levels compared to the controls

**Figure 2:**
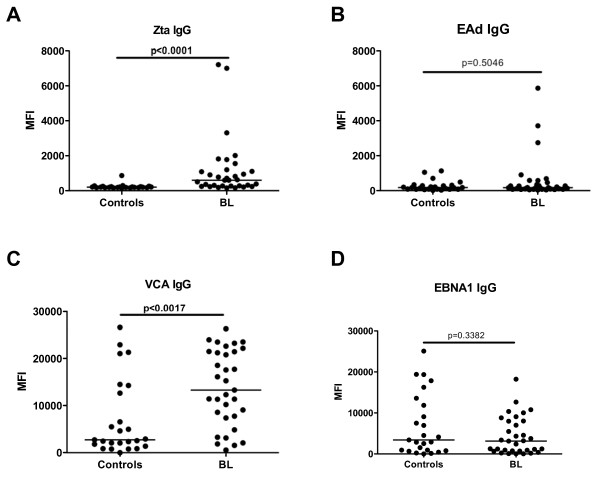
**EBV-specific IgG in controls compared to BL patients**. Plasma from BL patients (n = 32) and controls (n = 25) was diluted at 1:6400 and tested using a Luminex Bead Based array. Specific EBV antigens were (A) Zta-specific IgG, (B) EAd-specific IgG, (C) VCA-specific IgG, (D) EBNA1-specific IgG. The MFI of 75 of Luminex beads for each of the antigen tested is indicated on the y-axis. *P *values of Mann-Whitney U tests are indicated in the figures. Horizontal bars represent median values per each study population.

We next determined if there were differences in the EBV-specific IgG between BL patients and age-and gender-matched controls. We measured IgG specific for the EBV latent antigen, EBNA-1, and the EBV lytic antigens, Zta, EAd, and VCA using well characterized synthetic peptides as the target antigen in our multiplex assay [16,20,21]. We found significantly higher levels of Zta and VCA-specific IgG levels in BL patients compared to controls (*p *< 0.0001 and *p *= 0.0017, respectively), while the levels of EBNA-1 and EAd-specific IgG levels were comparable between the two groups (*p *= 0.3382 and *p *= 0.5046, respectively) (Fig. 2).

### Children with abdominal tumors have higher levels of Zta-specific IgG antibodies compared to children with jaw tumors

**Figure 3:**
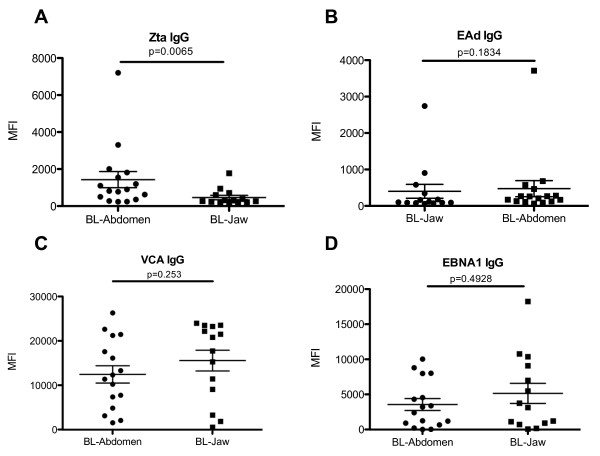
**EBV-specific IgG in BL patients with jaw tumors (n = 14) and abdominal tumors (n = 16)**. Plasma was diluted at 1:6400 and tested using a Luminex Bead Based array. Specific EBV antigens were (A) Zta-specific IgG, (B) EAd-specific IgG, (C) VCA-specific IgG, (D) EBNA1-specific IgG. The MFI of 75 of Luminex beads for each of the antigen tested is indicated on the y-axis. The differences between two study populations were compared using Mann-Whitney U test. *p *values of Mann-Whitney U tests are indicated in the figures. Horizontal bars represent median values per each study population.

To investigate if there were differences in the median levels of EBV-specific IgG antibodies among BL patients presenting with distinct clinical features, we stratified the study population into BL patients presenting exclusively with jaw tumors (n = 14) and those presenting exclusively with abdominal tumors (n = 16) and compared these to the controls (n = 25). The two patients presenting with both jaw and abdominal tumors and the patient presenting with a tumor in the lower limb were excluded from this analysis. We observed elevated levels of Zta-specific IgG antibodies in BL patients presenting with abdominal tumors compared to those presenting with jaw tumors (*p *= 0.0065). However, there were not any significant differences of VCA, EAd, or EBNA1-specific IgG between patients presenting with jaw tumors compared to those presenting with abdominal tumors (Fig. 3).

### EBV viral load in plasma is higher in patients with abdominal tumors compared to jaw tumors

**Figure 4:**
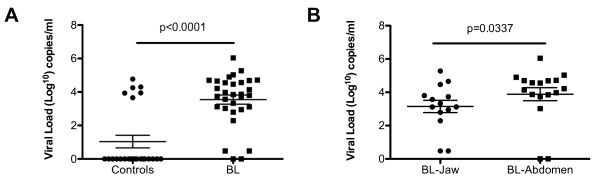
**EBV viral load in BL patients and controls**. PlasmaEBV viral loads were determined by Q-PCR and the values obtained were log-transformed. (A) The plasma EBV viral loads were significantly higher in BL patients (n = 32) compared to the controls (*p *< 0.0001), (B) Plasma EBV viral loads were significantly higher in patients presenting with abdominal tumors (n = 16) compared to jaw tumor patients (n = 14) (*p *< 0.0337). The differences between the two study populations were compared using Mann-Whitney U test. *p *values of Mann-Whitney U tests are indicated in the figures. Horizontal bars represent median values per each study population.

Other studies have identified elevated EBV viral loads in plasma from patients with nasopharyngeal carcinoma or EBV-associated Hodgkin's disease [22,23]. To determine if there were elevated plasma viral loads in BL patients relative to age-and gender-matched controls, we measured EBV levels in plasma by Q-PCR. As shown in Fig. 4A, EBV DNA was readily detected in the plasma in 30 out of 32 (94%) of BL patients. In contrast, only 6/25 (24%) of the controls had detectable levels of EBV in the plasma. Interestingly, the viral loads in the six control samples with detectable levels of EBV in the plasma were comparable to the levels in BL patients (medians, 4.105 log copies/ml versus 3.860 log copies/ml, respectively, *p *= 0.1018).

When we compared EBV viral load in plasma from BL patients with jaw tumors to BL patients with abdominal tumors, we observed significantly higher levels of EBV in plasma from BL patients presenting with abdominal tumors compared to those presenting with jaw tumors (*p *= 0.0337) (Fig. 4B). Elevated viral load in the plasma could be due to increased release of viral DNA from tumor cells due to apoptosis or necrosis of tumor cells, and not necessarily linked to elevated anti-Zta and VCA IgG levels. To determine if Zta and VCA IgG antibody levels correlated with EBV viral load, we did a Spearman correlation and found that Zta-specific IgG levels were positively correlated with plasma EBV viral loads in BL tumors (r = 0.3825, *p *= 0.0307) while there was no correlation with VCA IgG levels (r = -0.0972, *p *= 0.6093).

## Discussion

In our study, we set out to examine whether there was a difference in EBV antibody responses in BL patients with different clinical presentations, e.g. abdominal or jaw tumors. In addition, we wanted to examine whether there were elevated Zta antibody levels in BL patients relative to age-and gender-matched controls. We observed significantly higher levels of antibody against Zta IgG in all BL patients compared to malaria-exposed matched controls and significantly higher levels of Zta IgG in children with abdominal tumors compared to children with jaw tumors. These data suggest that the underlying clinical differences observed in site of BL presentation could be potentially related to differences in EBV biology.

A defining characteristic of patients with nasopharyngeal carcinoma (NPC), another EBV-associated malignancy, is elevated anti-Zta IgG and plasma DNA levels [24]. Our data extend these observations to BL, where we observed significantly elevated anti-Zta IgG as well as anti-VCA IgG and plasma viral loads in BL patients, relative to controls. Of note is that the elevated Zta antibody levels correlated with elevated viral DNA in the plasma. That both of these antibodies to EBV lytic antigens and viral DNA were high could be linked to increased viral re-activation in the tumors or to increased viral re-activation from latently-infected cells preceding the emergence of the malignancy. This latter possibility is supported by a previous study demonstrating that elevated anti-VCA antibodies in children precedes the emergence of BL [14]. The former is supported by evidence of EBV lytic gene expression in a subset of cells within endemic BL tumors [11,25-27] and, thus, could lead to elevated plasma viral loads following release of virus from lytically-infected cells. What is surprising is that there is no difference in levels of EAd antibodies between controls and patients suggesting that the correlation between elevated plasma EBV DNA and EBV lytic re-activation is complex. No significant differences in EBNA-1 antibody levels between cases and controls were observed, consistent with our previous study on EBNA-1 antibody levels in BL patients using an ELISA-based method [28].

Interestingly, differences in humoral immune responses against EBV have also been reported under a variety of different pathological conditions. For example, in HIV patients, anti-VCA IgG correlates with EBV viral loads, while in solid-organ transplant patients, viral load correlates with both anti-EA and VCA IgG titers [29,30]. Moreover, within a single EBV protein, i.e. Zta, different epitope responses have been described for different EBV-linked disease entities, such as infectious mononucleosis, NPC and non-Hodgkin lymphoma [31]. Further studies are needed to understand the nature of EBV persistence and re-activation in patients with EBV-associated malignancies to determine if there are underlying alterations in the pattern of EBV persistence that might precipitate or predict the emergence of a malignant clone.

We found that BL patients with abdominal tumors had higher levels of both anti-Zta IgG and plasma DNA levels compared to patients presenting with tumors in the jaw. Although the mechanisms that lead to the elevated anti-Zta IgG and plasma DNA levels in the abdominal tumor patients is still unclear, it could result from increased tumor burden in this group, which is indicated by the higher plasma LDH levels observed in these patients. In NPC, tumor burden has also been associated with elevated anti-Zta IgG and plasma DNA levels [32]. However, the question as to why there are also no concomitant increases in VCA or EAd IgG levels in the patients presenting with abdominal tumors, need to be addressed. In one study of BL, only mRNA from Zta could be detected in the tumors but not mRNA from another lytic transcript [25] suggesting that there is an abortive lytic cycle in the tumors.

Chronic exposure of children to *P. falciparum *malaria has been etiologically linked to Burkitt's lymphoma (reviewed in [3,4,14]). In support of these previous studies, two recent case control studies demonstrated that BL cases had elevated anti-malaria antibody titers compared to controls [8,15]. However, in contrast to these studies, we found no differences in IgG levels to a broad panel of pre-erythrocytic and erythrocytic antigens from the two common circulating *P. falciparum *strains (3D7 and FVO) in BL patients and controls. Discrepancies between these studies and our current findings may be attributed to differences in study design. In our study, we used controls from an area that has high incidence of BL and experiences persistent *P. falciparum *transmission [7], while in the previous studies, the controls were sick children presenting at the hospital with malignancies and non-malignant conditions and were from primarily urban areas as compared to the cases [8,15]. We chose a population-based control group because most children admitted to the Nyanza Provincial General Hospital for non-malignant illness are from Kisumu city whereas the children with BL are typically from rural regions within the Province [33]. This was true for our cohort as most of the BL patients in the current study were from rural settings. Thus, since both our cases and controls are from rural areas where malaria burden is higher than in urban areas, it is more likely that our cases and controls have similar exposure to *P. falciparum*. This could have, however, resulted in over-matching of cases to controls and limited our ability to identify differences in *P. falciparum *antibody responses. A larger population based case-control study is clearly needed to resolve the differences between our small study and the hospital case-control studies done in Uganda and Malawi [8,15].

## Conclusions

Children presenting with abdominal BL have elevated plasma EBV DNA, paralleled by elevated IgG levels to Zta, compared to children presenting with jaw tumors, suggesting differences in underlying patterns of EBV replication. Whether BL presenting in the abdomen is a different clinical entity will require more detailed studies of the tumors themselves.

## Methods

### Study population

During the period from August 2007 and September 2008, we prospectively enrolled 34 children presenting with endemic Burkitt's lymphoma at the Nyanza Provincial General Hospital, Kisumu, Kenya. Nyanza Provincial General Hospital is located in Kisumu city. As the largest hospital in the Province, it provides out-patient and in-patient services, and maintains its own pathology and radiology laboratories. The hospital is the referral center for childhood cancer cases and is the only medical facility that maintains chemotherapeutic treatment for BL in the region. The BL patients in this study predominantly came from all Districts in Nyanza Province (e.g. Siaya, Homa Bay, Kisumu, Migori, Kisii) as well as some Districts in Western Province (e.g. Kakamega, Vihiga, Nandi, and Busia). With the exception of Kisumu District which includes Kisumu City, these Districts are predominantly rural. BL diagnosis was based on histological assessment of fine needle aspirate (FNA) stained with May-Grunewald Giemsa. Assessment was done by both the clinical cytologist and pathologist at NPGH. One patient was diagnosed with acute leukemia and was excluded from further analysis. Routine HIV testing is done on all patients admitted to NPGH. HIV exposure was determined in venipuncture blood using two rapid serological assays: Unigold (Trinity Biotech, Bray, County Wicklow, Ireland) and Determine (Abbott Laboratories, Chicago, Illinois, USA). One BL patient was HIV+ and was excluded from further analysis. All blood samples were taken from these patients prior to chemotherapy.

Controls were identified from Kanyawegi village along the shores of Lake Victoria in Kisumu West district, Nyanza province, after physical and clinical evaluation of their health status by the study clinician officers. Kanyawegi is in a rural district that has a high incidence rate of BL and experiences persistent *P. falciparum *transmission [7]. After informed consent, blood samples were also collected from 25 healthy age-and gender-matched children (herein referred to as controls).

### Microscopic investigation of *P. falciparum *parasites

Parasitemia was determined at time of blood collection by performing a thick smear and staining with 5% Giemsa. The slides were examined by two microscopists, any discrepancies in the slide reading were resolved by a third microscopist. Parasite density was expressed as the number of asexual *P. falciparum *per *μL *of blood assuming a leukocyte count of 8000 per *μL*.

### Blood collection and processing

Ethical approval for this study was given by the Kenya Medical Research Institute (KEMRI) Ethical Review Committee, Institutional Review Board for Human studies at the Case Western Reserve University (Dr. Moormann's institutional affiliation at time of sample collection) and SUNY Upstate Medical University. After informed consent was given by the study participants' parent or guardians, finger prick blood was collected in EDTA tubes and measurements of hemoglobin levels were determined using a portable β-haemoglobin photometer (Hemocue AB Angelholm, Sweden). Additionally, 2-5 ml of venous blood was drawn from both BL patients and controls. Ficoll density gradient centrifugation of blood was done within 1 hour of blood collection; the plasma was removed for subsequent serologic or virologic analysis. The aliquots were then stored in a -80°C freezer. Biochemical analysis of lactate dehydrogenase (LDH) levels to determine tumor burden was carried out on frozen plasma samples using Selectra E auto analyzer (Vita lab, Amsterdam, The Netherlands) following standard biochemistry procedures using Fortress diagnostics kits (Fortress Diagnostics Limited, Belfast, UK).

### *P. falciparum *and EBV antigens

*P. falciparum*-specific IgG was detected using five of recombinant *P. falciparum *antigens, MSP-1(3D7), MSP-1 (FVO), AMA-1 (3D7), AMA-1 (FVO) and LSA-1 (in collaboration with Dr. Carole Long for MSP-1, Dr. David Lanar for AMA-1, and Dr. Sheetij Dupta for LSA-1) and EBV-specific IgG was determined using four synthetic peptides representing immuno-dominant epitopes of the viral capsid antigen (VCA), EBV nuclear antigen 1 (EBNA1), diffuse early antigen complex (EAd) and immediate early protein (Zta) antigens of EBV. The definition and serological use of these EBV peptide reagents has been described before [17,18]. In early studies we defined that the predominant response to Zta in BL patients was directed against the N-terminal domain, spanning amino acids 1-44 (Middeldorp, unpublished data). Therefore, this domain was used as synthetic Zta antigen throughout this study.

### Luminex assay

In order to detect *P. falciparum *and EBV-specific IgG against a panel of peptides, we used the previously described protocol [15]. Briefly, different amounts of peptides or proteins were coupled with 1 × 10^6 ^pre-activated carboxylated microspheres (Luminex, Austin TE, USA) in 500 μl of 100 mM MES pH 6.0 buffer (peptides) or 50 mM MES pH 5.0 buffer (proteins). The beads were then washed and stored in PBS, 0.1% BSA, 0.002% Tween-20, 0.05% Sodium Azide, pH 7.4 at 4°C until use. The amounts of peptides used per 500 μL coupling reaction were 20 μg for VCA, EBNA1 and Zta, and 50 μg was used for EAd. The amounts of recombinant malaria proteins per 500 μL reaction were 10 μg for the AMA-1 and MSP-1 antigens, and 20 μg for LSA-1. During the development of the assay, several sample dilutions were tested (ranging from 1:35 to 1:51,200) to determine the best dilution for testing IgG levels to different antigens in the test panel. It appeared that the Luminex assay was more sensitive than ELISA assays performed in parallel, and that in order for a majority of results to fall within the linear part of a standard curve (e.g. dilutions of a positive control sample versus fluorescent units) a dilution of 1:6400 was most appropriate. Therefore, we tested all samples at the typical 1:100 dilution as well as 1:6400. During the analysis, it appeared that indeed the 1:6400 dilution allowed for better distinction between high and low antibody levels than the 1:100 dilution. Thus, antigen specific IgG was measured by incubating 1000 beads of each antigen per well with plasma diluted 1:100 and 1:6400 in a final volume of 100 μl, but the results of the 1:6400 dilution were used in the final analysis. After washing, a 1:200 dilution of PE-conjugated Goat F(ab)_2 _anti Human IgG (Biosource, Camarillo, CA) was added. At least 75 beads of each antigen were then acquired on a Bioplex reader (Bio Rad, Hercules, CA). Sera from North Americans who had no prior exposure to *P. falciparum *were used as negative controls while pooled plasma from individuals who were constantly positive for *P. falciparum *antibodies were used as positive controls for *P. falciparum *antigens. In addition, EBV-seronegative and -seropositive controls were used in each plate. The results of the assay were expressed in Mean Fluorescent Intensity (MFI) of at least 75 beads for each *P. falciparum *and EBV antigen tested.

### Quantitative PCR to quantify EBV DNA

DNA was extracted from 200 μl plasma using Qiagen DNAeasy kit (Qiagen, Valencia, CA) according to the manufacturers' protocol. DNA was eluted off the column in an equivalent volume of H_2_0 and stored at -20°C. Previously designed primers and probes that detect a 70 bp region of the EBV BALF5 gene were used [34]. The quantitative (Q)-PCR cycle was as follows: 2 min at 50°C, 10 min at 95°C, 42 cycles of 15 sec at 95°C and 1 min at 60°C using thermal cycler model I Cycler™ optical module (BioRad Laboratories, Hercules, CA). IQ SuperMix (BioRad Laboratories, Hercules, CA) was used for all reactions. To generate a standard curve, we used an EBV positive plasmid generated from the PCR product. The viral loads were log-transformed and then calculated based on EBV genome copies/ml.

### Statistical analysis

GraphPad prism version 5 (GraphPad Software, Inc, La Jolla, CA) was used for all the data analysis. Differences in the levels of *P. falciparum *and EBV-specific IgG levels between controls and BL patients were compared using Mann-Whitney U test. The same test was used to compare both antigen-specific IgG levels and viral loads between children presenting with jaw and abdominal tumors. Across group comparisons of antigen-specific IgG levels and viral loads in controls, BL patients with jaw and abdominal tumors, was carried out using Kruskal-Wallis test. The association between categorical variables for healthy and BL patients was assessed using Fisher's exact test. Two-way ANOVA was used to evaluate the association between antigen-specific IgG levels with diseases status. Correlations between antigen specific IgG levels and viral load were done using Spearman's Rho test. *p *≤ 0.05 was considered statistically significant.

## Competing interests

The authors have no financial conflict of interest. JM is owner and CEO of Cyto-Barr BV, but declares no commercial interest in this study.

## Authors' contributions

AA, EP, AM and RR conceived of the study, participated in the study design and helped draft the manuscript. AA, EP, and PSO performed the study. NF performed PCR assays; JO provided clinical oversight; JMM, DEL, SD and CL provided peptides for Luminex assay and analysis of data from assays, and WGZ and CO provided supervision of AA and assisted with study design. All authors read and approved the final manuscript.
